# Methyl Angolensate from Callus of Indian Redwood Induces Cytotoxicity in Human Breast Cancer Cells

**Published:** 2010-09

**Authors:** Kishore K. Chiruvella, Kuppusamy Panjamurthy, Bibha Choudhary, Omana Joy, Sathees C. Raghavan

**Affiliations:** *Department of Biochemistry, Indian Institute of Science, Bangalore, India*

**Keywords:** double-strand breaks, intrinsic pathway of apoptosis, cancer therapeutics, alternative medicine, nonhomologous DNA end-joining

## Abstract

**AIM:**

Natural products discovered from medicinal plants have played an important role in the treatment of cancer. Methyl angolensate (MA), a tetranortriterpenoid obtained from the root callus of Indian Redwood tree, *Soymida febrifuga* Roxb. (A.Juss) was tested for its anticancer properties on breast cancer cells.

**METHODS:**

Cell viability was tested using trypan blue, MTT and LDH assays. Tritiated thymidine assay and flowcytometry were used to study effect of MA on cell proliferation. The activation of apoptosis was checked by annexin V and JC-1 staining followed by FACS analysis. Immunoblotting analysis was used for studying expression of apoptotic and DNA double strand break repair proteins.

**RESULTS:**

We find that MA inhibited the growth of breast cancer cell line, T47D in a time- and dose-dependent manner. MA treatment led to the inhibition of cell proliferation as detected by tritiated thymidine assay and flowcytometry. Further, MA treated cells exhibited typical apoptotic morphological changes and led to the accumulation of subG1 peak in cell cycle distribution. The induction of apoptosis was further confirmed both by annexin V staining and JC1 staining. We also find that MA activates MAP kinase pathway to induce apoptosis. Besides, we find a time dependent activation followed by degradation of DNA double-strand break repair proteins upon treatment with MA.

**CONCLUSION:**

These results suggest that MA induces cytotoxicity in breast cancer cells. Further, the altered expression of DSB repair proteins in MA treated cells may control the induction of apoptosis in these cancer cells.

## INTRODUCTION

Breast cancer is one of the most common cancers in the world and accounts for approximately 30% of all cancers in women. It also occurs in men but is about 100-fold less frequent than in women (1). Although the incidence of breast cancer has increased, the mortality rate has dropped during the last 10-15 years. The most dramatic decrease is seen in younger patients. Like numerous forms of cancer, breast tumor growth is a multifactorial disease with no standardized medication available. Different treatment modalities have been described against breast cancer, which include endocrine therapy, immunotherapy and chemotherapy. However, the major drawback of some of these treatments is their side effects, which deteriorates the quality of life of the patients. Lack of appropriate treatment is compounded by lack of efficacy and high toxicity of existing products. Hence, there remains a significant area of unmet medical needs. Therefore, although breast cancer research has developed at a rapid pace over the last decade, the curative potential of currently available therapies still needs to be improved.

Exploration of novel targets and strategies for the treatment of breast cancer is one of the active areas of research. There exist more than 270,000 higher plants, of which only a small portion has been explored for their phytochemical properties. It is anticipated that plants can provide potential bioactive compounds for the development of new ‘leads’ to combat cancer. Natural products or their derivatives comprised 14 of the top 35 drugs in 2000, based on worldwide studies (2). Chemoprevention, via ingestion of natural or synthetic agents with low toxicity that can suppress, delay, or reverse carcinogenesis, are being considered as a new dimension in the management of neoplasia (3, 4). Natural products have been shown to be excellent and reliable sources for the development of such new drugs. Triterpenoids are one of the families of natural compounds which are known for their medicinal value and they form a large and diverse class of organic compounds in plants. Methyl angolensate (MA) used in the present study is a tetranortriterpenoid. It is isolated from root callus of *Soymida febrifuga* (Meliaceae) and exhibits various biological effects (5-11). It is known to possess antimalarial, anti-inflammatory, antiallergic, antifungal, antiulcer, spasmolytic, insect antifeedant and phytoanticipin properties (5, 9, 12, 13). Earlier it has been shown that treatment with MA could induce apoptosis in leukemic and lymphoma cells (6, 14). Here we report that MA can induce cytotoxicity on breast cancer cells and trigger apoptosis. We also find that MA treatment led to the activation of MAP kinase pathway and induction of DNA double-strand break repair in a time-dependent manner.

## MATERIALS AND METHODS

### Chemicals and reagents

Unless otherwise mentioned, all the chemicals used were from Sigma-Aldrich, USA. Tritiated thymidine was purchased from BRIT, India. Annexin V-FITC and antibodies were purchased from Santa Cruz Biotechnology, USA.

### Cells and cell culture

ZR751 and T47D cell lines used in the present study were purchased from National Center for Cell Science, Pune, India. Cells were grown in RPMI 1640 (SERA LAB, USA) containing 10% FBS (GIBCO BRL, USA), 100 U of Penicillin G/ml and 100 μg of streptomycin/ml at 37°C in a humidified atmosphere containing 5% CO_2_. Cells were split in the ratio of 1:10 every 3-5 days.

### Isolation and purification of methyl angolensate (MA)

MA used in the present study was purified as described earlier (7). *Soymida febrifuga* Roxb. (A.Juss) plant used in the present study was collected from forests of Tirupathi, Andhra Pradesh, India. It was identified and authenticated with voucher specimen (SSR 1679) at SKU herbarium, India, recognized by Kew gardens, London, UK. Brown calli (600 g) obtained on MS medium supplemented with 2, 4-D alone and in combination with BA/2-iP from root explants of *Soymida febrifuga* were used in the present study. The calli was first dried at room temperature and crushed into a fine powder. The powder was subjected to soxhlation with hexane, ethyl acetate and methanol to get the soluble fraction. Ethyl acetate extract on concentration yielded a brown viscous residue (40 g). This residue was subjected to column chromatography over silica gel using hexane: ethyl acetate mixtures in increasing polarity. Hexane:ethyl acetate (7:3) elutes yielded fraction A, which was a mixture of compounds as observed using TLC. The mixture of compounds was further subjected to purification using silica gel (100-200 mesh) column. Hexane:ethyl acetate (8:2) fractions (21-30) on concentration gave a single spot on TLC, which was crystallised as colourless crystalline needles (1500 mg), and was designated as SF-1. SF-1 obtained as colourless needles (1500 mg) from methanol/chloroform, m.p. 203-205°C, was analyzed by LC-MS, which showed a molecular ion peak at m/z 470. It was further supported by ^13^C NMR spectrum, which showed signals for all the 34 carbons present in the molecule. Literature survey revealed that the physical and NMR spectral data of SF-1 were in good agreement with those recorded for methyl angolensate with the available data (7).

The purified MA was dissolved in DMF (Sigma, USA) and used for experiments. The maximum concentration of DMF (Dimethylformamide) used was equal to 0.05% and the same amount was used as vehicle control. In the experiments described herein MA was added to the cells after 24 h.

### Trypan blue exclusion assay

Trypan blue assay was carried out to assess the effect of MA on the viability of ZR751 and T47D cells as described (15). Briefly, the cells were seeded, at a density of 0.5 × 10^5^ cells/ml in complete medium. Following 24 h of cell growth, different concentrations of MA (10, 100, or 250 μM) or vehicle (DMF) were added to the cells. After 48 and 96 h, cells were trypsinized, washed and resuspended in PBS containing 0.4% trypan blue. The number of viable cells were counted using haemocytometer as per standard protocol. Each experiment was done a minimum of three independent times with good agreement.

### MTT assay

Cytotoxic effect of MA on ZR751 and T47D cells was measured by using MTT assay (16). In brief, cells were treated with MA (10, 100, or 250 μM) as described above, harvested after 48 and 96 h and MTT reagent (5 mg/ml) was added and incubated for 4 h. The absorbance of samples was measured after incubation in SDS (10%) for 2 h using a microplate reader (Molecular Devices, USA) scanning spectrophotometer at a wavelength of 570 nm. Cells grown in appropriate concentrations of DMF were used as control. Absorbance is a function of concentration of soluble yellowish MTT to the insoluble purple formazan, which is formed by active mitochondrial dehydrogenase in living cells. The cell proliferation was calculated as a ratio of absorbance of sample to the absorbance of untreated control.

### [^3^H]-Thymidine incorporation assay

T47D cells were cultured in duplicates in 96 well plates in a volume of 0.125 ml (0.5 × 10^5^ cells/ml) and treated with MA as described above. An aqueous solution of [^3^H] thymidine (1 μCi) diluted with RPMI 1640 was added to each well after 8 h of treatment with MA as described earlier (16). After 48 and 96 h of incubation, the cells were trypsinized, pelleted and washed in PBS multiple times at 4°C. The pellet was suspended in 5% TCA for 30 min and centrifuged. Again the pellet was resuspended in 50 μl of ice cold PBS, loaded on a filter paper disc (Sartorius, Germany) and dried overnight at 37°C. Discs were transferred to scintillation liquid and radioactive counts were taken on a liquid scintillation beta-counter. Radioactive counts per minute (cpm) was recorded and percentage of inhibition of proliferation was calculated considering the cpm of untreated cells as 100% and represented as a bar diagram.

### LDH release assay

LDH assay was performed to assess the LDH release to the media following MA treatment (50, 100 and 250 μM) on T47D cells for 24, 48 and 72 h as per standard protocols (17). The intracellular LDH was determined after lysing the cells by freezing and rapid thawing. The LDH release was measured at an absorbance of 490 nm. The percentage of LDH release was calculated as: (LDH activity in media)/(LDH activity in media + intracellular LDH activity)×100.

### Cell cycle analysis by flow cytometry

T47D cells were cultured and treated with different concentrations of MA. After 72 h of treatment, cells were harvested, washed and permeabilized using 70% ethanol at -80°C overnight. The fixed cells were centrifuged at 5,000 rpm, washed twice and resuspended in ice cold PBS. RNase (10 mg/ml) was added and incubated overnight at 37°C. Ethidium bromide (EtBr, 50 μg/ml) was added half an hour before acquiring the flow cytometer reading (FACScan, BD Biosciences, USA). A minimum of 10,000 cells were acquired per sample and bar diagrams were analyzed by using WinMDI 2.8 software.

### Annexin V-FITC/propidium iodide (PI) staining assay and confocal microscopy

The frequencies of apoptotic cells were detected with the annexin V-FITC apoptosis detection kit (Santacruz, USA). T47D cells were cultured for 72 h with MA, (100 and 250 μM), collected, washed and resuspended in 1x binding buffer (HEPES-NaOH 10 mM pH7.4, 1.4 M NaCl and 25 mM CaCl_2_) at a concentration of 1 × 10^6^ cells/ml. Annexin V-FITC (0.2 μg /μl) and PI (0.05 μg/μl) were added and incubated in dark for 10 min. Cells were then subjected to FACS analysis. At least 10,000 events were recorded and represented as dot plots.

Confocal microscopy was performed to visualize the apoptotic cells generated as a result of treatment with MA. Annexin V-FITC and PI (0.05 μg/μl) staining was done as described above. The cells were then spread over a microscopic slide, mounted using cover slip and viewed with an inverted confocal laser scanning microscope (Ziess LSM 510 MK4, Germany).

### Detection of mitochondrial membrane potential (Δ*Ψm*)

The mitochondrial membrane potential (Δ*Ψ*m) was analyzed using JC-1 (5,5’,6,6’ tetrachloro-1,1’3,3’ tetraethylbenzimidazolcarbocyanine iodide; Calbiochem, USA) (18), a lipophilic cationic fluorescent dye capable of selectively entering mitochondria and acting as a dual emission probe that reversibly changes color from green (FL-1) to greenish orange (FL-2) as the mitochondrial membrane becomes more polarized (18). Briefly, cells were harvested, washed, resuspended in PBS and incubated with JC-1 (0.5 μM) at 37°C for 15 min. Cells were then washed in PBS and analyzed by flow cytometry using CellQuest Pro software at an excitation of 488 nm laser and emission at 530/30, 580/610 nm. JC-1 monomers emit at 530 nm (FL-1 channel) and J-aggregates emit at 590 nm (FL-2 channel). 10,000 cells were acquired per sample. 2, 4-Dinitrophenol-treated cells (2,4-DNP; positive control) were used for compensation (19).

### Immunoblotting

Cell lysates were prepared from MA treated T47D cells (100 μM for 24, 48 and 72 h or, 10, 25, 50, 75, 100 μM for 72 h). For immunoblot analysis, 20 μg of protein was loaded on a SDS-PAGE (8-10%), transferred to PVDF membrane (Millipore, USA) and probed with appropriate primary and secondary antibodies (Santacruz, USA). The primary antibodies used are KU70/80, p53, DNAPKcs, pERK½, pMEK½, Caspase 3, PARP, BAD (Santacruz, USA), NBS1, RAD50 and MRE11 (BD Biosciences Pharmingen). The blot was developed using chemiluminescent solution (Immobilon ^TM^ western, Millipore, USA) and bands were detected by chemiluminescence system (LAS 3000, FUJI, JAPAN). Blots were stripped subsequently as per standard protocol and reprobed with anti-tubulin, which served as a loading control.

Quantitation of bands in the western was carried out using ImageQuant software. We first selected a rectangle area covering the protein band in one lane and quantified the intensity. Then the same sized rectangle was placed on other bands of interest and quantified in each lane. An equal area from the same lane in the blot where there was no specific band, was used as background and its intensity was subtracted from that of other bands. The intensity of the bands was then plotted and presented as a bar diagram.

### Statistical analysis

The results are expressed as the mean plus or minus standard error. All analyses were performed with the GraphPad software using one-way ANOVA followed by Tukey-Kramer Multiple Comparison Test. Statistical significance was considered at *p*<0.05.

## RESULTS

### MA induces cytotoxicity in a concentration-dependent manner

**Figure 1:**
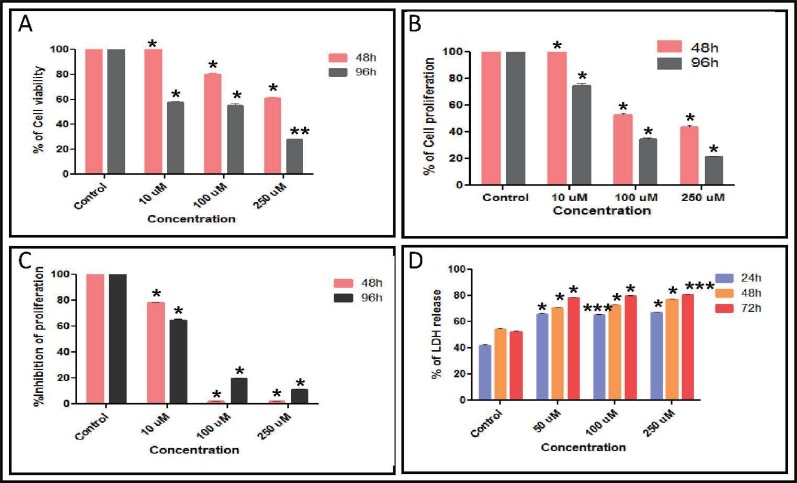
Analysis of cytotoxicity induced by methyl angolensate on breast cancer cells. **A,** Determination of cell viability after treating T47D with MA. Human breast cancer cell line, T47D was seeded into 6 well culture plates (0.5 × 10^5^ cells/ml). MA was added after 24 h at concentrations of 10, 100 and 250 μM. DMF was used as vehicle control. The cell viability was measured by the intake of trypan blue by dead cells. Live cells were counted at 48 and 96 h and the data was represented as a bar diagram. **B,** Determination of cell proliferation by MTT assay. T47D cells were cultured with 10, 100 and 250 μM of MA for 48 and 96 h. After harvesting the cells, MTT (5 mg/ml) was added and processed as described in Methods. The absorbance at 570 nm was measured using ELISA plate reader and plotted as bar diagram. **C,** [^3^H]-thymidine incorporation assay to determine effect of MA on cell proliferation. MA (10, 100 or 250 μM) was added to T47D (0.5 × 10^5^/ml) cells after 24 h of culture. After 8 h of addition of MA, aliquots of the culture were transferred to 96 well plates in duplicates to which tritiated thymidine (1 μCi) was added. Cells were harvested after 48 and 96 h and radioactive counts per minute (cpm) was calculated and percentage of inhibition of proliferation was calculated considering the cpm in untreated as 100% and plotted as a bar diagram. **D,** Measurement of LDH release following treatment with MA. After the exposure of T47D cells with MA at different concentrations (50, 100 and 250 μM) for 24, 48 and 72 h, the release of LDH was measured at 490 nm. Results are presented as percentage of LDH release. The % of cell viability and inhibition of cell proliferation was calculated considering control cells as 100%. In all the cases, error bars indicate SD from three independent experiments. P values (*p*<0.05) denoted by asterisk were calculated comparing mean control cells with mean treated cells (*p<0.05, **p<0.1, ***p<0.5).

**Figure 2:**
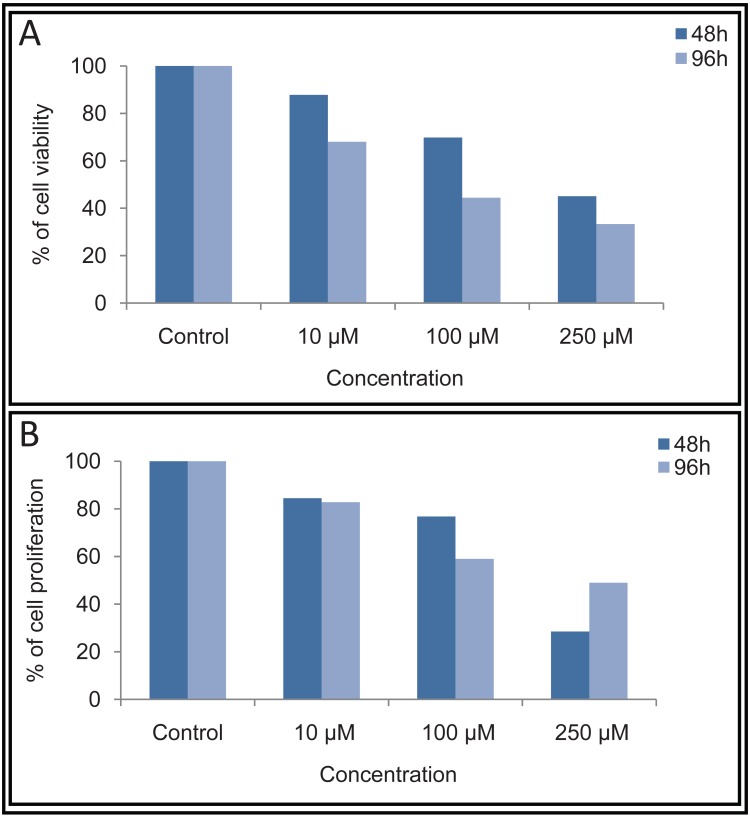
Determination of cell viability and cell proliferation by trypan blue and MTT assay following MA treatment on ZR751 cells. Cells were cultured with 10, 100 and 250 μM of MA or DMF control for 48 and 96 h. The trypan blue and MTT assay was performed as described in Fig. 1 legend. The % of cell viability and cell proliferation was calculated considering untreated as 100% and plotted.

In the present study, MA was tested for its cytotoxicity on two breast cancer cell lines, ZR751 and T47D using trypan blue and MTT assays. Cells growing in log phase were treated with 10, 100 or 250 μM concentrations of MA. As a vehicle control, cells were also treated with 0.05% DMF (equivalent to DMF concentration in highest dose of MA). In order to evaluate the cytotoxic effect of MA on growth of breast cancer cells, we have used trypan blue assay. The cells were counted at 48 and 96 h of treatment with MA after staining with trypan blue. The effect was limited when 10 μM MA was used. However, concentrations of 100 and 250 μM resulted in increased cell death (Fig. 1A and Fig. 2A). These results suggest that MA induces cytotoxicity in human breast cancer cells in a dose- and time-dependent manner.

The cytotoxicity induced by MA on proliferation of ZR751 and T47D cells was further verified using MTT assay. Cells treated with 10, 100 and 250 μM of MA were harvested after 48 or 96 h and were subjected to MTT assay. Results showed that cell proliferation was affected upon treatment with MA at concentrations of 100 and 250 μM (Fig. 1B and Fig. 2B).

However, there was no significant effect at 10 μM as seen by trypan blue assay as well. Results also showed that the cell death induced by MA was much more pronounced after 96 h of MA treatment (Fig. 1B). These results further suggest that MA induces cytotoxicity in breast cancer cells. Based on the above results, the IC50 of MA for T47D was estimated to be approximately 90 μM. Since ZR751 had higher IC50 for MA than T47D and was comparatively insensitive to MA, we have not used it for further studies.

Since above studies suggested that MA affects the viability of the T47D cells, we were interested to know whether MA could disrupt DNA synthesis and thereby cell division or it just induced cell death directly by activating apoptotic pathways. To test this, we cultured the T47D cells in the presence of [^3^H] thymidine following the addition of MA as described in “Materials and Methods”. Results showed that upon addition of MA (100 and 250 μM), the incorporation of [^3^H] thymidine reduced dramatically compared to the controls (Fig. 1C). We could find a reduction in incorporation of [3H] thymidine even after treating with 10 μM of MA (Fig. 1C). These results suggest that MA affects the cell viability by inhibiting cell division probably by interfering with DNA replication. However, it is also possible that in addition to its effect on cell division, it could induce apoptosis. Further, LDH release assay also showed a dose- and time-dependent increase in the LDH release upon treatment with MA on T47D, further confirming the above results (Fig. 1D). However, when a normal lymphoblastoid cell line, GM00558B was analyzed by MTT assay following MA treatment, we could not find any significant effect on cell survival (14).

### Flow cytometry analysis showing induction of apoptosis by MA

**Figure 3:**
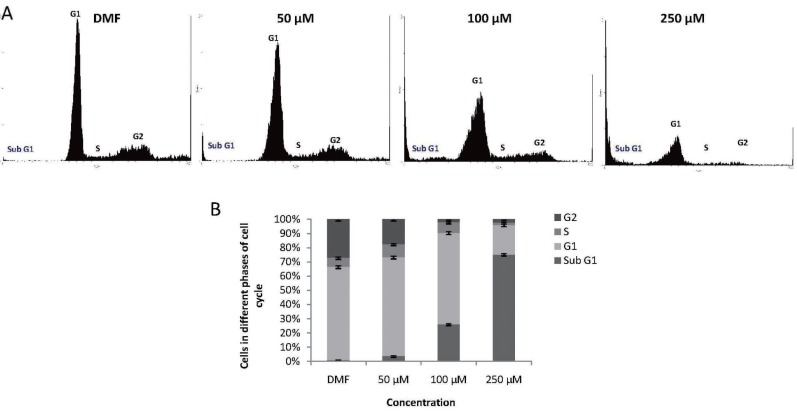
Cell cycle analysis of breast cancer cells following MA treatment. **A,** Breast cancer cells (T47D) were grown in the presence of different concentrations of MA (50,100 and 250 μM) or appropriate concentration of DMF (vehicle control) for 72 h. The cells were harvested, stained with ethidium bromide and DNA content was quantified by flow cytometry. **B,** Histogram showing the percentage of cells in the G0/G1, G1, S and G2/M phase of the cell cycle obtained after FACS analysis. For each sample 10,000 cells were acquired. Each experiment was repeated three times and values are the mean of three replicates ± SE.

Since [^3^H] thymidine incorporation assay suggested an effect of MA on T47D cell division, we performed FACS analysis to determine the effect of MA on cell cycle progression. Interestingly upon addition of MA, a concentration dependent change was observed in the cell cycle pattern (Fig. 3). Cell cycle analysis showed that 50, 100 and 250 μM, MA treatment in T47D cells resulted in a concentration dependent accumulation of cells in subG1 population, which indicated apoptotic cells, as compared to control (Fig. 3B). In non-apoptotic population, the portion of cells in G1 and S phase remained same when the cells were treated with 50 and 100 μM, however a drastic decrease of cells in G2/M phase was noted suggesting that cell cycle progression may be blocked. When a concentration of 250 μM was used, most of the cells accumulated in sub-G1 phase indicating apoptosis (Fig. 3B). These findings indicate that at the range of concentrations studied, the anti-proliferative effect of MA on T47D cells could be attributed to a block in cell cycle progression followed by induction of apoptosis. Thus, the results obtained, further confirmed our earlier observations that MA could interfere in cell division. We also find accumulation of subG1 population of cells in a concentration dependent manner, which is a hall mark of apoptosis.

### Evaluation of apoptosis by annexin V-FITC staining

**Figure 4:**
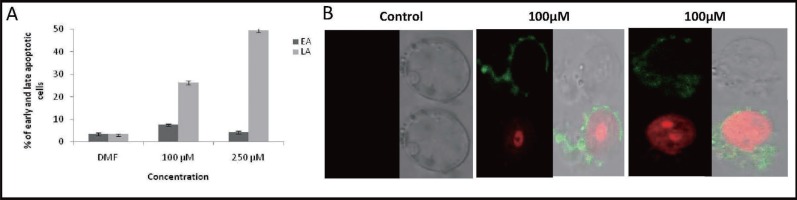
Detection of apoptosis induced by MA in breast cancer cells using annexin VFITC/PI staining. **A,** T47D cells were cultured with DMF alone (vehicle control), 100 μM or 250 μM of MA for 72 h and processed for annexin V-FITC/ PI double-staining. Quantification of cells undergoing apoptosis was carried out by flow cytometry. The early and late apoptotic cells from each panel is quantified and presented as a bar diagram. EA- early apoptosis, LA- late apoptosis. Both JC-1 aggregates and monomers exhibit fluorescence in the green end of the spectrum which is measured in the Green (FL-1) channel while only JC-1 aggregates show a red spectral shift resulting in higher levels of red fluorescence emission which is measured in the Red (FL-2) channel. Each experiment was repeated three times and values are the mean of three replicates ± SE. **B.** Visualization of apoptotic cells by confocal microscopy. The MA treated T47D or respective vehicle controls cells were annexin V-FITC/PI double-stained as described above. Microscopic evaluation of the stained cells showed that most of the control cells were negative for both annexin V-FITC and PI, indicating live cells with cellular integrity, indicated as “Control” (left panel). Depending on the extent of apoptosis, cells were stained differently. Cells in the early apoptotic stage stained only with annexin V (membrane damage) and appeared green in color. Cells in the late stage of apoptosis were stained with both annexin V and PI and appeared in both green and red color indicated as “100 μM” (middle and right panel).

Since we could observe accumulation of cells at G0/G1 peak during cell cycle analysis following treatment with MA, we were interested in quantifying the different types of apoptotic cells. Phosphatidyl serine residues, which are normally located in the internal phospholipid layer are actively translocated to the external layer in apoptotic cells and thus can be detected by annexin-V staining. In order to detect and quantify the apoptosis induced by MA, we have used annexin V-FITC/PI double staining. T47D cells were harvested after 72 h of treatment with MA (100 and 250 μM) and used for double staining followed by FACS analysis. Dot plot results showed a time- and dose-dependent increase in late apoptotic cells, which is represented as a bar diagram (Fig. 4A). Although there was an increase in early apoptotic cells the effect was less prominent. MA induced effect showed that at a concentration of 100 μM, about 7.54% of cells were in early apoptotic stage (stained only by annexin V-FITC) (Fig. 4A), while about 26.25% of the cells were in late apoptotic stage (stained by annexin V-FITC and PI), which was 8.5 times higher than that of DMF control (Fig. 4A). At a concentration of 250 μM this was observed to be 4.19% and 49.98% (15.2 times higher than control), respectively (Fig. 4A). These results suggest that MA induces apoptosis in a dose-dependent manner. The annexin V/PI double stained cells indicate that in such cells extensive cell membrane damage has occurred, which results in nuclear staining. These conclusions were further verified qualitatively by observing the above stained cells under a confocal microscope. The results showed that control cells (DMF treated) were negative for annexin V-FITC and PI. At 100 μM concentration of MA we could see cells stained by both annexin V-FITC (green colour) and PI (red color) suggesting complete damage of cell membrane (Fig. 4B). These results suggest a total disruption of cell membrane and further damage to the chromosomal DNA upon treatment with MA.

### MA induces mitochondrial depolarization

**Figure 5:**
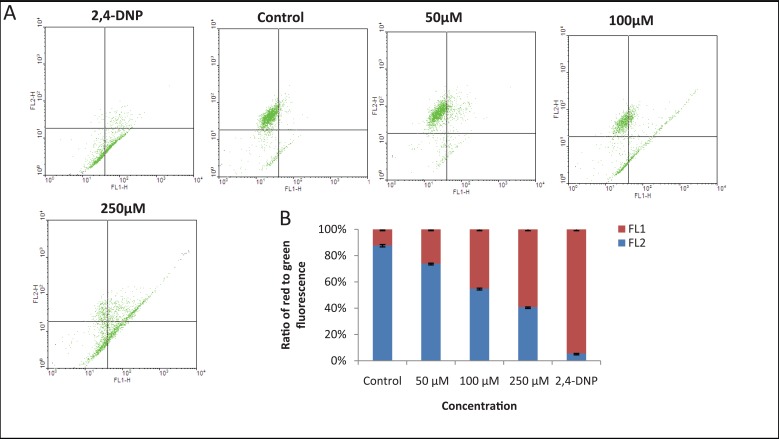
Effect of MA on mitochondrial transmembrane permeability transition (ΔΨm). T47D cells incubated with different concentrations of MA for 72 h were stained by JC-1 dye and loss of mitochondrial membrane potential was assessed with the signal from monomeric and J-aggregate JC-1 fluorescence by flow cytometry. DMF treated cells are taken as control. 2,4DNP was used as a positive control. **A,** Dot plot represents an increase in ΔΨm with increase in concentration of MA. **B,** Bar diagram showing ratio of apoptotic (FL1) and non-apoptotic cells (FL2). Each experiment was repeated three times and values are the mean of three replicates ± SE.

Mitochondrial membrane depolarization is an early event of apoptosis, which increases the mitochondrial transmembrane potential (MTP) and facilitates the release of pro-apoptotic factors, including cytochrome c, AIF, SMAC and other, as yet unidentified, factors into the cytosol (20, 21). Using the mitochondria specific probe, JC-1, a lipophilic cationic fluorescent dye with dual emission wavelengths, we tested the effect of MA on mitochondrial depolarization in T47D cells. Elevated mitochondrial transmembrane potential has been shown to increase JC1 fluorescence at 530 nm (FL1-H), corresponding to its monomeric form, and to reduce JC-1 fluorescence at 590 nm, corresponding to its dimeric form. Consequently, mitochondrial depolarization is indicated by a decrease in the red/green fluorescence intensity ratio. Results showed that DMF treated cells, mostly exhibited red fluorescence indicating an intact mitochondrial transmembrane potential (Fig. 5). Upon addition of MA, the number of cells showing green fluorescence increased in a concentration dependent manner, compared to positive control 2,4-DNP (Fig. 5A, B). This suggests that addition of MA disrupted the MTP in a concentration-dependent manner resulting in cytosolic accumulation of monomeric JC-1.

### MA treatment regulates expression of DNA double-strand break repair proteins at early time points

Since the above results demonstrated the activation of apoptosis in MA treated cells, we were interested to see whether MA could induce DNA strand breakage. In order to test this, T47D cells were treated with 50,100 and 250 μM of MA for 72 h, harvested and used for extraction of chromosomal DNA. The results showed fragmentation of the DNA leading to a smear, although it was to a limited extent (data not shown). The observed smear could be due to DNA breakage at multiple positions across the chromosomal DNA. These results indicate that MA is able to induce DNA damage leading to DSBs.

**Figure 6:**
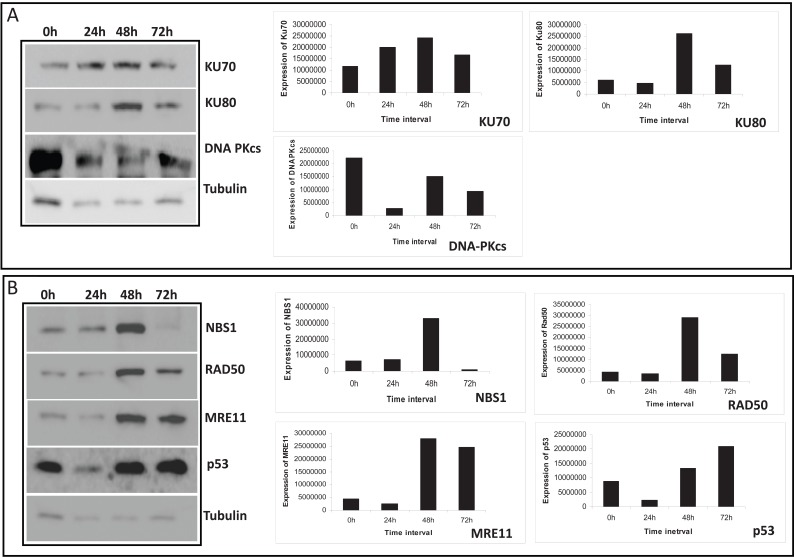
Treatment of MA leads to the upregulation of enzymes associated with DNA repair. Cell lysate was prepared from T47D cells after treating with 100 μM of MA for 24, 48 or 72 h. DMF treated cells grown for 72 h were used as control. Immunoblotting was performed as described in Methods. The α-tubulin was used as an internal loading control. **A,** The time dependent expression of KU70, KU80 and DNA-PKcs are studied. **B,** Time dependent expression pattern of NBS1, RAD50, MRE11 and p53 proteins. The bar diagrams showing quantification of respective gels are shown on the right.

Generally, damage to cellular DNA should lead to activation of repair proteins, so that the breaks could be repaired. There are two different pathways in human cells to repair DNA double–strand breaks (DSBs); nonhomologous DNA end joining (NHEJ) and homologous recombination (HR) (22, 23). Among these, NHEJ is the major DSB repair pathway in humans. Since we have seen a dose-dependent increase in DNA strand breaks following treatment with MA, we were interested in assessing the status of NHEJ proteins in treated cells. To test this, we performed western blot analysis after preparing the cell lysates from MA treated T47D cells (100 μM after 24, 48 and 72 h). KU70 and KU80 are DNA end binding proteins involved in NHEJ that bind to the ends of the DNA once breaks occur. Western blot analyses have showed that upon treatment with MA, the levels of KU70 and KU80 increased for first two days (upto 48 h). However, treatment upto 72 h led to the downregulation of KU proteins (Fig. 6A). This is understandable as the amount of cytotoxicity was limited upto 48 h of MA treatment. In addition, we noted that the expression of DNAPKcs, the kinase involved in NHEJ, reduced, as the time of incubation increased following treatment with MA (Fig. 6A). Hence, it appears that following MA treatment, first the cellular machinery tries to repair the DSBs, however, prolonged treatment leads to the accumulation of strand breaks which becomes irreparable, leading to the activation of apoptosis.

The protein p53, is known as the guardian of the genome. Earlier studies have shown that following treatment with anticancer agents, normally the expression of p53 gets upregulated. In the present study we note that treatment with MA led to the significant upregulation of p53 from 48 h onwards (Fig. 6B). The MRN complex is another nuclease involved in both HR and NHEJ. Our results show that following treatment with MA, its expression was remarkably increased at 48 h (Fig. 6B). However, further incubation upto 72 h, led to downregulation of all these proteins. It is important to point out that in all these cases expression of MRN complex remained same after 24 h of MA treatment (Fig. 6B). Therefore, our results suggest that expression of DNA DSB repair proteins was significantly regulated upon treatment with MA.

**Figure 7:**
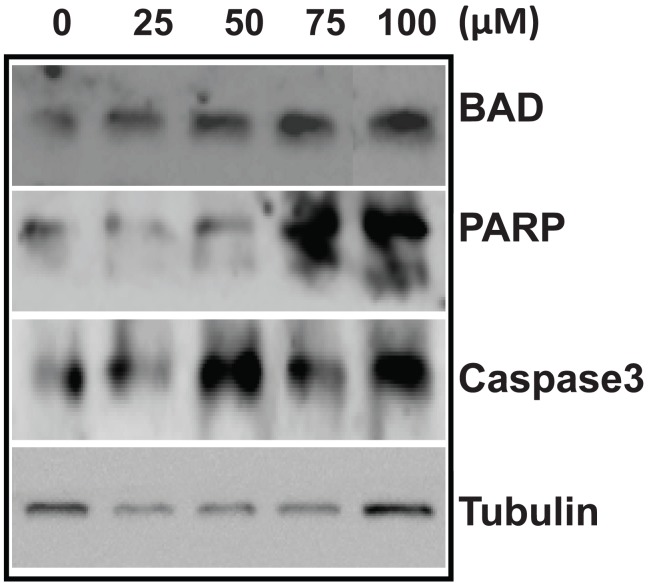
Expression profile of apoptotic proteins in T47D following MA treatment. Cell lysate was prepared from T47D cells after treating with 0, 25, 50, 75 and 100 μM of MA for 72 h. DMF treated cells grown for 72 h was used as control. Immunoblotting was performed as described in Methods. The α-tubulin was used as an internal loading control. Dose dependent expression pattern of apoptotic proteins studied are BAD, PARP and Caspase 3. In all the panels “0” is DMF control; 25, 50, 75 and 100 μM of MA are cells harvested after 72 h of MA treatment.

Results show that BAD expression was upregulated, in a dose-dependent manner (Fig. 7). Poly (ADP-ribosyl) polymerase (PARP) is a single strand break repair enzyme known to be cleaved by caspase 3. Upon immunoblotting using anti-PARP, we found a 89 kDa band corresponding to the cleaved product at 75 and 100 μM (Fig. 7) after 72 h of treatment. Since we could see PARP cleavage, we were interested in testing the expression of caspases 3. Our results showed activation of procaspase 3, (Fig. 7). Since the antibody used against procaspase 3, could not recognize its cleaved product we were unable to detect the cleaved caspase 3. Reprobing with anti-tubulin antibody, the loading control, confirmed that the amount of protein loaded was mostly comparable in all experiments (Fig. 7). Therefore, immunoblot analysis suggests that MA induces apoptotic proteins in T47D. Activation of caspase 3 followed by PARP cleavage suggest that MA might be inducing the intrinsic apoptosis pathway to trigger cell death.

### Mitogen activated protein kinase regulation by methyl angolensate

**Figure 8:**
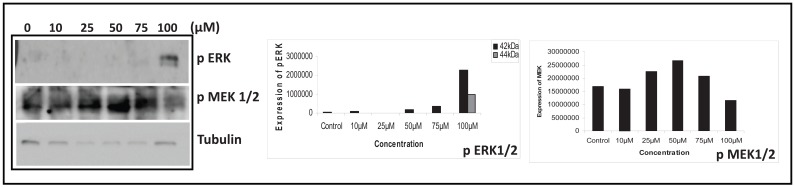
MA activate MAPK pathway to induce apoptosis. Cell lysate was prepared from T47D cells after culturing with 0, 10, 25, 50, 75 and 100 μM of MA for 72 h. Deregulation of signaling molecules was studied by immunoblotting analysis. Concentration dependent expression pattern of MAPK signaling proteins studied are pERK and pMEK1/2. The α-tubulin was used as an internal loading control. In all panels, “0” is DMF control; 10, 25, 50, 75 and 100 μM are the concentrations of MA used. The bar diagram showing quantification of respective gels are shown on the right.

To investigate the molecular signals involved during apoptosis induced by MA, we studied the activation status of the MAP kinase family proteins, since they are well known to be involved in the control of a variety of cell survival-controlling pathways (24). This was accomplished by using antibodies against phosphorylated (activated) forms of ERK1/2, and MAPK/JNK and determining the status of these proteins in control and MA-treated cells (10-100 μM) by immunoblotting. Results showed that treatment with MA led to phosphorylation of JNK, in a concentration dependent manner, 75 μM showing maximum activation (data not shown). Interestingly, unlike JNK, phosphorylation of ERK was detectable only at 100 μM of MA (Fig. 8). In the case of MEK1/2 we could see a concentration dependent activation only upto 50 μM. Further increase did not show any phosphorylation (Fig. 8). Immunoblot analysis of phosphop38 proteins revealed increased level of phospho-p38, which was also detected only at 100 μM of MA concentration (data not shown). These findings indicate that treatment with MA led to the activation of JNK, ERK and p38 in breast cancer cells.

## DISCUSSION

Majority of cancer patients respond to radiation and chemotherapy at the earlier stages of cancer therapy. However, once cancer reaches to metastasis, generally tumors fail to respond to conventional treatment. Such patients often seek help in complementary or alternative medicines. A large variety of herbal products are available as dietary supplements for cancer patients. Unfortunately, very little is known about their efficacy and mode of action, and possible adverse interactions with conventional anticancer drugs. Therefore, testing the medicinal plant products *in vitro* on cancer cells to understand their mechanism of action is helpful in enhancing their effectiveness.

Various terpenoids are attractive natural compounds, which are used as therapeutic agents for the treatment of cancer (25, 26). The ability to induce apoptosis in tumor cells is an important property of a candidate anticancer drug. It becomes even better when the molecule can discriminate between cancer cells and normal cells (27). The search for natural products from *Soymida febrifuga* based on ethnobotanical considerations has led to the isolation of methyl angolensate from root callus (23). Two cell lines ZR751 and T47D were used in the present study to test the sensitivity of MA on breast cancer. Results suggest that MA is more sensitive for T47D than ZR751 cell line. The inhibition of proliferation by MA observed was different against different kinds of cancer cells, indicating that methyl angolensate have relatively a wide spectrum of antitumour activity (6, 14).

In the present study, we find that MA induced cytotoxicity by activating apoptosis in the breast cancer cell line, T47D. Although MA could induce cytotoxicity in these cells, the IC50 value was about 90 μM, which is higher than many synthetic compounds. This is not surprising as methyl angolensate is a natural product. In fact, previous studies have also used similar doses for other compounds (28-31).

Our results showed that MA affected growth of breast cancer cells in a time-and dose-dependent manner. Tritiated thymidine assay and FACS analysis showed that MA interferes with cell division leading to inhibition of cell proliferation ultimately triggering to cell death. Generally cell death could be either through necrosis or apoptosis. The former process is associated with relatively large damage to the surrounding tissue and latter with controlled elimination of cancer cells. Thus for the possible treatment of breast cancer, cytotoxic compounds should preferentially act via apoptosis. To address the question whether methyl angolensate induces cell death via apoptosis, we used different cellular assays. Using various assays, we find that MA treatment led to the induction of apoptosis in breast cancer cells which was seen by enhanced loss of plasma membrane polarity as detected by Annexin V staining (Fig. 4), sub-G1 phase accumulation as indicated by FACS analysis (Fig. 3) and loss of mitochondrial transmembrane potential observed by JC1 staining (Fig. 5) and DNA fragmentation (data not shown). In this respect, it is important to point out that many studies have been performed earlier showing that compounds from plants, activate apoptosis in different cancer cells (26, 32).

If MA mediated cell death is through the intrinsic pathway of apoptosis, one would anticipate a change in the mitochondrial transmembrane potential leading to the release of cytochrome c. In agreement with this, we have shown here that in T47D cells, MA decreases mitochondrial membrane potential. Indeed, mitochondria are now thought to act as key coordinators of apoptosis (33). While the inner mitochondrial membrane (IMM) remains relatively intact, the outer mitochondrial membrane (OMM) becomes completely permeabilized to proteins such as pro-caspases 2, 3, and 9, cytochrome c, AIF, SMAC, Endo G etc, resulting in their leakage from the mitochondrial intermembrane space (21).

In a series of investigations to search for anti-cancer agents from plant sources, terpenoids exhibited an anti-proliferative property by inducing apoptosis and targeting mitochondria with a decreased membrane potential, leading to the activation of the intrinsic apoptotic signal transduction (34, 35). Our studies using JC-1 staining indicated a loss of mitochondrial membrane potential (MMP), hence supporting the activation of intrinsic apoptotic pathway. Terpenoids have also been shown to induce apoptosis by activation of the intrinsic pathway (36). The current study indicates that treatment with MA displayed disrupted mitochondrial membrane potential resulting in activation of intrinsic pathway of apoptosis. Further, the observed activation of BAD, Caspase 3, PARP supports such a hypothesis. Similar activation of mitochondria mediated apoptosis has also been shown by MA in leukemic cells (6).

MA treatment led to the activation of various DNA repair genes related to DNA double-strand break repair in a time dependent manner. We find that the KU proteins involved in NHEJ were upregulated immediately after the treatment with MA. KU70 and KU80 are two DNA end binding proteins which are normally involved in the protection of the ends following induction of a DSB. It has been suggested that KU might regulate the apoptotic cell death by acting as an inhibitor of apoptosis. In contrast degradation of KU may also help in preventing the inappropriate repair of fragmented nuclear DNA during apoptosis (37). Consistent to this we noted that prolonged incubation with MA resulted in degradation of the KU protein. Similar results were obtained in bleomycin, vincristine or adriamycin treated HeLa cells (38). We also noted that the kinase involved in NHEJ, DNAPKcs was also down regulated upon MA treatment.

Thus, our observation suggests that upon MA treatment, the extent of breaks generated was initially limited and therefore the NHEJ machinery was activated. However, prolonged treatment led to accumulation of DNA damage which were beyond the level of repair by the cellular machinery. This led to the downregulation of NHEJ proteins including MRN nuclease suggesting that probably the extent of damage induced was too high, and beyond the limit of the repair machinery to act upon. In contrast to this current observation, our previous study on the effect of MA on leukemic cells had shown a complete downregulation of the NHEJ proteins.

A growth inhibitory signal could be generated by the p53 tumor suppresser, a protein that is induced by DNA damage caused by cytotoxic agents or radiation (39). Consistent to this, our data clearly indicates that treatment with MA on breast cancer cells activates expression of p53 after 48 h of treatment. Similar induction was also seen when eurycomanone was treated on MCF7 cells (40).

Cellular commitment to apoptosis, or the ability to evade apoptosis in response to damage, involves the integration of a complex network of survival and death pathways. Antitumor agents, despite having diverse primary mechanisms of action, mediate their effects by inducing apoptosis in tumor cells. MAPK signalling pathways have been implicated in the response of tumor cells to chemotherapeutic drugs. While the activities of the major MAPK subgroups are subject to modulation upon exposure of different types of cancer cell lines to diverse classes of antitumor agents, the response tends to be context-dependent, and can differ depending on the system and conditions (41). Various phytochemicals have been shown to modulate the signaling pathways of MAPKs, leading to growth inhibition and cell death (42, 43). We found that MA increased the amount of phosphorylated ERK1/2 in T47D cells, suggesting that this pathway was activated in these cells and may have triggered the apoptosis, although ERK1/2 activation exerts a cytoprotective effect (44). Therefore our studies suggest that MA could activate MAPK pathways to induce apoptosis in breast cancer cells. Such findings necessitate additional *in vitro* and *in vivo* studies for investigating the role of MA against breast cancers. However chemical synthesis always plays an important role in production of terpenoid drugs and hence synthesis of MA can be used for studying structure–activity relationships (SAR). However, unfortunately in the literature till date, using MA as a backbone, no novel molecules are synthesised or elucidated. Hence further studies are needed to derive new cytotoxic molecules with low IC50 for treating various cancers.

## References

[1] Cooper GM (1992). Elements of human cancer.

[2] Butler MS (2004). The role of natural product chemistry in drug discovery. J. Nat. Prod.

[3] Aziz MH, Kumar R, Ahmad N (2003). Cancer chemoprevention by resveratrol: *in vitro* and *in vivo* studies and the underlying mechanisms (review). Int. J. Oncol.

[4] Gupta S, Ahmad N, Mukhtar H (1999). Prostate cancer chemoprevention by green tea. Semin. Urol. Oncol.

[5] Amos A (2002). Behavioural effects in rodents of methyl angolensate: a tetranortripernoid isolated from Entandrophragma angolense. Basic Clin. Pharm. Toxicol.

[6] Chiruvella KK (2008). Methyl angolensate, a natural tetranortriterpenoid induces intrinsic apoptotic pathway in leukemic cells. FEBS Lett.

[7] Chiruvella KK (2007). Phytochemical and Antimicrobial Studies of Methyl Angolensate and Luteolin -7 -O-glucoside Isolated from Callus Cultures of Soymida febrifuga. Int. J. Biomed. Sci.

[8] Njar VC, Adesanwo JK, Raji Y (1995). Methyl angolensate: the antiulcer agent of the stem bark of Entandrophragma angolense. Planta. Med.

[9] Orisadipe A (2001). Spasmolytic activity of methyl angolensate: a triterpenoid isolated from Entandrophragma angolense. Biol. Pharm. Bull.

[10] Bickii J (2000). *In vitro* antimalarial activity of limonoids from Khaya grandifoliola C.D.C. (Meliaceae). J. Ethnopharmacol.

[11] Thioune O, Pousset JL, Lo I (1994). Anti-inflammatory activity of the bark of Khaya senegalensis (A.Juss.). Preliminary research of structure and activity relationship. Dakar Med.

[12] Abdelgaleil SA, Hashinaga F, Nakatani M (2005). Antifungal activity of limonoids from Khaya ivorensis. Pest Manag. Sci.

[13] Penido C (2006). Inhibition of allergen-induced eosinophil recruitment by natural tetranortriterpenoids is mediated by the suppression of IL-5, CCL11/eotaxin and NFkappaB activation. Int. Immunopharmacol.

[14] Chiruvella KK, Raghavan SC (2010). A natural compound, methyl angolensate, induces mitochondrial pathway of apoptosis in Daudi cells. Invest New Drugs.

[15] Shahabuddin MS (2009). A novel DNA intercalator, butylamino-pyrimido[4’,5’:4,5]selenolo(2,3-b)quinoline, induces cell cycle arrest and apoptosis in leukemic cells. Invest New Drugs.

[16] Kavitha CV (2009). Novel derivatives of spirohydantoin induce growth inhibition followed by apoptosis in leukemia cells. Biochem. Pharmacol.

[17] Korzeniewski C, Callewaert DM (1983). An enzyme-release assay for natural cytotoxicity. J. Immunol. Methods.

[18] Cossarizza A (1993). A new method for the cytofluorimetric analysis of mitochondrial membrane potential using the J-aggregate forming lipophilic cation 5,5’,6,6’-tetrachloro-1,1’,3,3’-tetraethylbenzimidazolcarbocyanine iodide (JC-1). Biochem. Biophys. Res. Commun.

[19] Han YH (2008). 2,4-dinitrophenol induces G1 phase arrest and apoptosis in human pulmonary adenocarcinoma Calu-6 cells. Toxicol *In Vitro*.

[20] Cande C (2002). Apoptosis-inducing factor (AIF): key to the conserved caspase-independent pathways of cell death?. J. Cell Sci.

[21] Ravagnan L, Roumier T, Kroemer G (2002). Mitochondria, the killer organelles and their weapons. J. Cell Physiol.

[22] Lieber MR, Yu K, Raghavan SC (2006). Roles of nonhomologous DNA end joining, V(D)J recombination, and class switch recombination in chromosomal translocations. DNA Repair (Amst).

[23] Chiruvella KK (2007). Mechanism of DNA Double-Strand Break Repair. The ICFAI J of Biotech.

[24] Versteeg HH (2000). The role of phosphatidylinositide-3-kinase in basal mitogen-activated protein kinase activity and cell survival. FEBS Lett.

[25] Castrillo A (2001). Inhibition of the nuclear factor kappa B (NF-kappa B) pathway by tetracyclic kaurene diterpenes in macrophages. Specific effects on NF-kappa B-inducing kinase activity and on the coordinate activation of ERK and p38 MAPK. J. Biol. Chem.

[26] Lee JH (2002). Kaurane diterpene, kamebakaurin, inhibits NF-kappa B by directly targeting the DNA-binding activity of p50 and blocks the expression of antiapoptotic NF-kappa B target genes. J. Biol. Chem.

[27] Frankfurt OS, Krishan A (2003). Apoptosis-based drug screening and detection of selective toxicity to cancer cells. Anticancer Drugs.

[28] Liu LF (2004). Action of solamargine on human lung cancer cells-enhancement of the susceptibility of cancer cells to TNFs. FEBS Lett.

[29] Reyes FJ (2006). (2Alpha, 3beta)-2,3-dihydroxyolean-12-en-28-oic acid, a new natural triterpene from Olea europea, induces caspase dependent apoptosis selectively in colon adenocarcinoma cells. FEBS Lett.

[30] Roy HK (2001). Polyethylene glycol induces apoptosis in HT-29 cells: potential mechanism for chemoprevention of colon cancer. FEBS Lett.

[31] Kumar A (2008). Growth inhibition and induction of apoptosis in MCF-7 breast cancer cells by a new series of substituted-1,3,4-oxadiazole derivatives. Invest New Drugs.

[32] Tan ML (2005). Methanolic extract of Pereskia bleo (Kunth) DC. (Cactaceae) induces apoptosis in breast carcinoma, T47-D cell line. J. Ethnopharmacol.

[33] Green DR, Reed JC (1998). Mitochondria and apoptosis. Science.

[34] Nakagawa Y (2005). A potent apoptosis-inducing activity of a sesquiterpene lactone, arucanolide, in HL60 cells: a crucial role of apoptosis-inducing factor. J. Pharmacol. Sci.

[35] Ohguchi K (2005). Vaticanol C-induced cell death is associated with inhibition of pro-survival signaling in HL60 human leukemia cell line. Biosci. Biotechnol. Biochem.

[36] Ikai T (2006). Magnolol-induced apoptosis is mediated via the intrinsic pathway with release of AIF from mitochondria in U937 cells. Biol. Pharm. Bull.

[37] Ajmani AK (1995). Absence of autoantigen Ku in mature human neutrophils and human promyelocytic leukemia line (HL-60) cells and lymphocytes undergoing apoptosis. J. Exp. Med.

[38] Kim SH (1999). Ku autoantigen affects the susceptibility to anticancer drugs. Cancer Res.

[39] Clark LJ, MacKenzie K, Parkinson EK (1993). Elevated levels of the p53 tumour suppressor protein in the basal layer of recurrent laryngeal papillomas. Clin. Otolaryngol. Allied Sci.

[40] Mahfudh N, Lope Pihie A (2008). Eurycomanone Induces Apoptosis through the Up-Regulation of p53 in Human Cervical Carcinoma Cells. J. Cancer Mol.

[41] Schaeffer HJ, Weber MJ (1999). Mitogen-activated protein kinases: specific messages from ubiquitous messengers. Mol. Cell Biol.

[42] Sah JF (2004). Epigallocatechin-3-gallate inhibits epidermal growth factor receptor signaling pathway. Evidence for direct inhibition of ERK1/2 and AKT kinases. J. Biol. Chem.

[43] Aggarwal BB (2004). Role of resveratrol in prevention and therapy of cancer: preclinical and clinical studies. Anticancer Res.

[44] Kang CD (2000). The inhibition of ERK/MAPK not the activation of JNK/SAPK is primarily required to induce apoptosis in chronic myelogenous leukemic K562 cells. Leuk. Res.

